# Comprehensive assessment of the physical and health features of the threatened Araguaian River dolphin *Inia araguaiaensis*

**DOI:** 10.1371/journal.pone.0319212

**Published:** 2025-03-31

**Authors:** Daniela M. D. de Mello, Waleska Gravena, Aricia Duarte-Benvenuto, Alan S. Lima, Fernando R. Gomes, Vera M. F. da Silva

**Affiliations:** 1 Department of Physiology, Institute of Biosciences, University of São Paulo, São Paulo, Brazil; 2 Associação Amigos do Peixe-Boi, Manaus, Amazonas, Brasil; 3 Laboratory of Wildlife Comparative Pathology - University of Sao Paulo, São Paulo, Brazil; 4 Instituto de Saúde e Biotecnologia, Universidade Federal do Amazonas, Coari, Amazonas, Brazil; 5 Laboratório de Mamíferos Aquáticos, Instituto Nacional de Pesquisas da Amazonia, Manaus, Amazonas, Brazil; Museu Paraense Emilio Goeldi, BRAZIL

## Abstract

The Araguaia River dolphin is endemic to the Tocantins-Araguaia River Basin and is under severe anthropogenic threats. Given the species’ vulnerability and historical neglect, preliminary data on its health and physical parameters are presented, along with an evaluation of potential differences between individuals from areas with varying human activity. A comparison of these data with its closely related species, the Amazon River dolphin *I. geoffrensis*, was also aimed. Twenty-four dolphins were captured and released in Cantão State Park (protected area, n = 10), Tocantins state; and Luiz Alves (exposed to fishing activities, n = 14), Goiás state. Preliminary data indicates no significant differences in the body morphology and physical parameters between individuals from the two areas. Araguaia River dolphins tended to show larger grey areas in their bodies than their counterparts, Amazon river dolphins. Cardiac rate, respiratory frequency, and oral temperature did not vary between individuals from different areas, sex, or age classes. Hematological and serum chemistry variables differences were observed among age classes, mainly related to body and immune system development. Dolphins from Cantão had higher AST and urea/creatinine and lower GGT, total bilirubin, and creatinine than dolphins from Luiz Alves. Such variations may reflect different prey types, intensities of muscle extenuation during capture, or subclinical diseases. Most hematological parameters for the Araguaia River dolphin were outside the reference intervals described for the Amazon river dolphin but inside its minimum and maximum ranges. Conversely, the serum chemistry values from both species overlap with the reference values. The highest serum testosterone was detected in adult males and the highest serum progesterone and estradiol in adult females. Correlations between reproductive hormones and body measurements demonstrated preliminary hormonal variation according to the body’s development as the individuals reach sexual maturity. Understanding the current physical and health status of individuals from areas with different human activity intensities is crucial to drive management efforts toward species conservation.

## Introduction

River dolphins are particularly vulnerable to environmental changes caused by direct or indirect human activities, and thus, are considered one of the most threatened groups of cetaceans. This could likely be explained by the restricted area where they inhabit, especially when compared to oceanic dolphins, and the escalating threats the freshwater ecosystems are facing [1]. For instance, the construction of Hydroelectric Power Plants (HPP), boat traffic, and intensive interaction with fishing activities increase the impacts caused by human activity [2,3]. Furthermore, extreme environmental conditions like droughts linked with El-Niño Southern Oscillation can reduce the water level and increase its temperature, which was recently associated with the death of hundreds of dolphins in Brazil [4]. The pumping of water for agricultural purposes can also culminate with the isolation of river dolphins, which become more prone to agonistic human interactions such as physical aggression and gunshots, and low food availability, which could ultimately lead to starvation and death [3,5,6]

The Araguaia River dolphin (*Inia araguaiaensis*) is a relatively recently described species of freshwater dolphin from Brazil; however, its classification as a single species is not a consensus. The taxonomic separation of the genus *Inia* within the Brazilian territory, comprising *I. geoffrensis* and *I. araguaiaensis*, was officially recognized by the Brazilian government in 2019 [7], and both species are currently classified as endangered by the Official List of Brazilian Flora and Fauna Species Threatened with Extinction [8]. The Amazon river dolphin (*I. geoffrensis*) inhabits the rivers of the Amazon Basin, drained by the Amazon River and its tributaries with water flowing from west to east [9]. Its counterpart, the Araguaia River dolphin, is found in the Tocantins-Araguaia River Basin which drains an area of biome transition between the Cerrado (Brazilian Savannah) and the Amazon forest, running from south to north and is located entirely in Brazil [10]. Hybrids of these two species were found in the Amazon Delta in the confluence of the Amazon and Tocantins-Araguaia Basin [11]. The 2600 km of the Araguaia River margins are occupied by miscellaneous anthropogenic activities such as intense agriculture, mining, and fishing intercalated with some protected areas along its course [12].

Despite its conservation status and cumulative threats, currently, there is no regular monitoring of the Araguaia River dolphin populations and their health. Some preliminary studies on the species density and distribution demonstrated that dolphins are more numerous in areas with lower human occupancy and boat traffic [13,14]. However, there is limited information on their basic physical and physiological characteristics or the potential impacts of human activities on their health. Investigations of the biological features and health assessments of free-ranging cetaceans, particularly threatened species, are crucial for the appropriate conservation actions [15,16]. Of note, studies on the critically endangered Yangtze finless porpoise (*Neophocaena asiaeorientalis* ssp. *asiaeorientalis*) inhabiting the Poyang Lake (China), have demonstrated how the increase of heavy vessel trafﬁc and dredging can be detrimental to the individual’s health leading to altered endocrinological, hematological and serum chemistry parameters [15]. However, before assessing such information, baseline values encompassing different aspects of the organism’s functioning (e.g., physical, endocrinological, vital parameters) should be determined to identify potential health alterations in a free-ranging cetacean population.

Investigations of the basic body size and shape of a recently described threatened species are crucial for the appropriate conservation actions. Distinct morphological and physical aspects may be identified among close species or even between/among different populations of the same species. For example, shape differences in the skulls of dolphins of the genus *Sotalia* were identified between marine and freshwater specimens which were later identified as two different species by genetic analysis [17,18]. The freshwater species (*S. fluviatilis*) is currently classified as endangered by the IUCN Red List, while the marine species (*S. guianensis*) is classified as near threatened [19,20].

Health monitoring of free-ranging cetaceans has also proven to be an efficient tool to identify potential threats and take conservation actions toward a determined population. Previous studies demonstrated that individuals of different life history stages and/or from distinct geographical locations can significantly differ in their hematological and serum chemistry parameters [21–23]. The number of leucocytes tends to be higher in immature individuals of Amazon river dolphins and Yangtze finless porpoises [21,23], as it occurs for most mammals. Serum elements such as calcium and alkaline phosphate activity were also higher in immature individuals due to bone formation [21,23].

Hormone levels from dolphins from different locations and/or under different levels of human activities also tend to vary [24]. Yangtze finless porpoises inhabiting an area with intense underwater noise presented statistically significant higher levels of serum cortisol, triiodothyronine (fT3), thyroxine (fT4), and lowered testosterone when compared to porpoises inhabiting a semi-natural reserve with lower human impacts [15]. Elevated blubber cortisol was detected in the Indo-Pacific humpback dolphins (*Sousa chinensis*) as an effect of contaminant exposure and changes in the availability of food resources in the Pearl River estuary, South China Sea [25].

**Fig 1:**
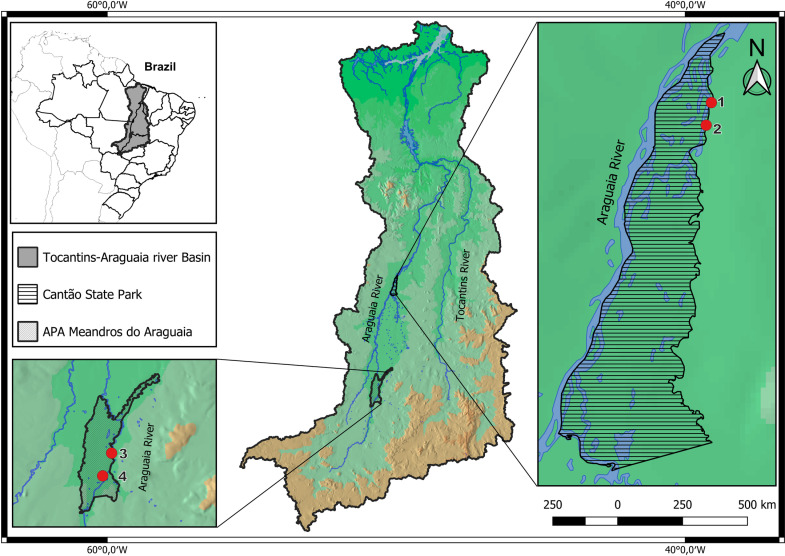
Tocantins-Araguaia River Basin in Central Brazil. Red dots 1 and 2 represent the capture-release sites of Araguaia River dolphins at Cantão State Park (n =  10), and red dots 3 and 4 show the capture-release sites in the APA Meandros do Araguaia (n =  14).

The Araguaia River basin spans two Brazilian biomes, the Cerrado and the Amazon, encompassing over 386,000 km² of territory. It is spread across 204 municipalities located within the states of Goiás, Mato Grosso, Tocantins, and Pará (Fig 1). Most of its area overlaps with human activities highly impacted by pesticide contamination from agriculture, deforested margins by several farms along its course, water pumping for agriculture irrigation, high boat traffic, water contamination by sewage, trace elements, and other chemicals [5,19,20,26–28]. Also, the intense tourism during the dry season, the sportive fisheries, and the intentional killing of dolphins during most of the year in areas such as Luiz Alves, located on the border of the Environmental Protected Area (APA) Meandros do Araguaia, Goiás state, represent threats to the species [2,13]. Meanwhile, conservation units, such as Cantão State Park, represent only 9.42% of its territory [19,29]. Usually, dolphins inhabiting highly impacted areas tend to have poorer health conditions than animals living in low human activity areas [30]. Until now, it remains unknown how these anthropogenic activities may affect the distribution and health of dolphin populations along the Araguaia River. In this scenario, the present study aims to provide preliminary insights into the Araguaia River dolphin’s physical, health, and endocrine aspects considering potential differences among age class or sex. These parameters are also compared between individuals in two different segments of the Araguaia River under different human activity intensities: Cantão State Park – a protected area, and Luiz Alves which is exposed to sport fishing activities and more intense boat traffic. Finally, some of the described information was compared to published reference data of its counterpart species the Amazon river dolphin [22,23].

## Materials and methods

### Area of study

The study was performed in two segments of the Araguaia River. Many segments and lakes of the river system remain partially or fully isolated during the dry season (July – November) representing a natural intermittent physical barrier with annual flood seasonality [31]. The first selected region for assessing the dolphins between 8 – 12 Sep 2022 was Cantão State Park, located in the Tocantins state, comprising an area of 890 km^2^ (Fig 1). The area is bordered in the southwest by the Bananal Island, along both the Araguaia and Javaés rivers [32]. The two sites chosen to capture the dolphins in that region were selected based on the favorable environmental conditions, including shallow and calm waters, usually inside a small bay, and depending on the constant or persistent presence of the dolphins.

The second region was located ~ 580 km downstream of the Cantão State Park in the vicinity of the municipality of Luiz Alvez, Goiás state. Luiz Alves is just on the Environmental Protected Area (APA) Meandros do Araguaia border. Despite the APA’s mainland being protected by law, most sections of the river and its tributaries are open for tourist activities. Intense boat traffic and sport fisheries occur in the area year-round [13]. The dolphins were captured between 15 – 19 Sep 2022 in two of the few locations of the APA where tourism is prohibited (Fig 1). Sampling sites were chosen based on the success probability of capturing the dolphins safely.

### Animal handling

A team of experienced field assistants used two boats and several nets to block and isolate small groups of animals in the predetermined area. Dolphins were captured using previous techniques designed for cetaceans in the Amazon basin [33]. The nets were 12 meters high and could completely block the passage of the individuals underneath them. Smaller nets were used to carefully drag the dolphin to the riverbank. Only one dolphin was handled at a time. The total time of capture and handling was registered in minutes, from the moment the animals were encircled with the small mesh net to their release after the sampling procedures.

The dolphins were placed on a sponge mattress in a shadowed area. Behavior, stress level, and physical parameters (e.g., respiratory frequency and cardiac rate) were closely monitored to ensure the safety of the dolphins, as detailed below [22]. Age classes were determined by observing the total body length and external features, including body color and the presence of scars [34]. Lactating females accompanied by a young individual were assigned as adults and calves, respectively. The sex was identified by the observation of the genital slit.

This work was carried out accordingly to a Federal-level permit by the Brazilian National Environmental Agency (IBAMA) SISBIO number 81758-1. Tocantins and Goiás state permits were granted by Instituto Natureza do Estado do Tocantins (NATURATINS) Nº 2022/40319/068696 and Secretaria de Estado de Meio Ambiente e Desenvolvimento Sustentável (SEMAD) Nº50/2022 - SEMAD/GEUPUC-18334, respectively. The study complies with the criteria for ethics and animal welfare ethics and animal welfare criteria from the Committee on the Ethics of Animal Experiments of the National Institute of Amazonian Research. Every effort was made to ensure the safety and well-being of both animals and personnel throughout the capture, examination, and blood sampling process. Our study did not involve the use of anesthesia, euthanasia, or surgical procedures. All animals were safely released after the sampling procedures with no intercurrence.

### Body aspects and morphology

The color of individuals was determined by visual inspection. Total body length (BL) was determined using a measuring tape in cm after positioning the dolphin straight on top of the mattress. The measure was done in centimeters from the cranial-most part of the rostrum to the final portion of the caudal fluke in a straight line. The maximum girth (MG) was taken by placing the measuring tape around the widest part of the body. The relation of the MG to BL (MG:BL) and of the weight (W) to BL (W:BL) were also calculated. A mobile pole structure with a hanging digital scale was used to determine the dolphin’s weight in kilograms.

### Physical parameters

The vital physical parameters of all dolphins were monitored. Respiratory frequency was determined by the number of openings in the blowhole for one minute. Cardiac rate was obtained with a stethoscope and the number of beats per minute was recorded. A digital thermometer (TD 911, ICEL Manaus, Brazil, resolution of 0.1˚C between 0˚ to 199.9˚C) was used to measure the oral temperature (OT). A flexible gauge was placed at the buccal cavity of the dolphins, between the teeth and the chick, for 15 seconds. The stress level was assigned following the criteria established for the Amazon river dolphin as (1) unstressed or no perceptible signs of stress; (2) some responsiveness to external stimuli and little resistance to handling and restraint; (3) moderate responsiveness to external stimuli and mild resistance to handling and immobilization; (4) extreme responsiveness to external stimuli and high resistance to handling and immobilization, frequent vocalization, temporary apnea [22].

### Blood analysis

Around 20 ml of blood was drawn from the ventral fluke vasculature after proper cleaning disinfection of the area, using a 19-gauge, 1.9 cm long butterfly catheter connected with 10-20 ml syringes. The blood was transferred to 5 ml EDTA tubes for hematological analysis and to 8.5 ml tubes with clot activator and polymer gel for serum chemistry and hormone analysis (Vacutainer SST II Advance, Becton Dickinson Indústrias Cirúrgicas Ltda., São Paulo, São Paulo, 04717–004, Brazil). After collection, the samples were immediately placed in a cooler with ice packs.

The blood cell analysis was performed using manual hematological techniques following conventional techniques for mammals within 8 h of blood collection [22,35]. Briefly, hematocrit (HCT) was determined by centrifuging a microcapillary filled with blood at 11,360 ×  g for 10 min and reading on a microhematocrit card (EZ reader; LW Scientific, Lawrenceville, GA). The hemoglobin concentration (Hb) was measured after placing 20 **μl** of whole blood into 5 mL of hemoglobin kit solution (Labtest Diagnóstica, Lagoa Santa, MG, Brazil) with a spectrophotometer at a wavelength of 540 nm. To avoid bias, a single trained individual did cell counting for all samples. Red blood cells (RBCs), white blood cells (WBCs), and platelets were counted using a Neubauer chamber with a light microscope at a magnification of 400X. The WBC differential count was performed on one Wright-stained slide per animal. One hundred WBCs were counted under 1,000X total magnification, and the percentage of each cell type was recorded to calculate total cell counts.

The clot activator and gel-containing tubes were centrifuged for 10 min at 4.400 X g for serum separation within 8 h of collection. The serum was stored for 10-21 days in a − 20°C freezer until biochemical analysis. Serum albumin, total proteins, globulins, calcium, glucose, cholesterol, triglycerides, phosphorus, magnesium, total bilirubin, direct bilirubin, indirect bilirubin, creatinine, urea, uric acid, phosphate; and enzyme activity of alkaline phosphatase (ALP), alanine aminotransferase (ALT), amylase, lipase, aspartate transaminase (AST), creatin kinase (CK), and game-glutamyl-transferase (GGT) were determined for all individuals. Biochemical analyses were performed using the automated equipment Dimension EXL 200 (Siemens Healthcare Diagnostics, Delaware, USA) and commercial Siemens Dimension® diagnostic kits (Siemens Healthcare Diagnostics, Tarrytown, NY, USA) for 21 analytes. Labtest kits (Labtest Diagnostica SA, Lagoa Santa, Minas Gerais, Brazil) were used to measure serum albumin and GGT.

### Hormone analysis

Serum samples were kept frozen at -80˚C before the hormone analyses. The assays were performed for progesterone, testosterone, estradiol, and cortisol using colorimetric enzyme immunoassay from the same manufacturer (Arbor Assays, Ann Arbor, MI). Steroid hormones were initially extracted with Diethyl Ether (https://www.arborassays.com/wp-content/uploads/2022/02/Steroid-Liquid-Extraction-190308.pdf) and determined by ELISA commercial kits (Cortisol #K003-H; Testosterone #K032-H; Progesterone #K025-H; Estradiol #K030-H Arbor ELISA kit), according to the manufacturer’s instructions. The assay for progesterone, testosterone, and cortisol followed manufacturer protocols without modifications. The assays for estradiol were modified by not diluting the serum before the assay. Total activity, non-specific binding wells, blanks (zero doses), and standards were included in all plates. All samples, standards, and controls were assayed in duplicate. Samples that eventually exhibited results with > 10% CV between wells were measured again. Assay parallelism was checked for all hormones with an *F*-test to compare the slopes of the linear portion of the standard curves and the serial dilutions of a pool of serum extract to test if the antibody binds well to the immunoreactive component in the sample of interest [36]. Accuracy was tested by the observation of the resulting dose of the pooled extract vs. the known standard dose with linear regression, with acceptable accuracy deﬁned as r^2^ >  0.95 and slope within the range of 0.7–1.3 [36,37].

### Statistical analyses

The means, medians, standard deviations (SD), minimum (min), and maximum (max) values were estimated for the physical and health parameters. Normality and homoscedasticity of variances were accessed before comparing two or more groups, or the correlations between two variables with the Shapiro-Wilk test and Levene’s test, respectively. The Student *t*-test was used to compare two groups (*e.g.,* males *versus* females) with normal distributed homoscedastic data and the Mann-Whitney test for non-normal heteroscedastic data. Calves and juveniles were grouped as “immature” for comparison purposes. The comparison of three groups or more was done using One-way Analysis of Variance (ANOVA) when variances were homoscedastic and with the Kruskal-Wallis when variances were not heteroscedastic. The Tukey post hoc test was used to verify significant differences among groups for comparisons with homoscedastic variances, while Dun-Bonferroni was used for comparisons with heteroscedastic variances. Differences among groups of all analyses were considered significant when the *P* value was <  0.05. Pearson correlation coefficient (r) was used to verify the potential relation between two variables for normally distributed data, and with Spearman’s rank correlation coefficient (r_s_) when data were not normally distributed.

Linear regression of observed dose versus known standard dose was used to assess the accuracy of all hormones. The F test was employed to evaluate the parallelism between the kit’s standard curve and the data obtained using diluted serum pools in assays for all hormones. Acceptable results were considered when differences between standard and diluted serum pool curves were not significant (*P* >  0.05).

Statistical analyses were performed with XLSTAT Software version 2023.2.1414 (Addinsoft, Paris, France), Microsoft Excel®, and GraphPad Prism version 10.1.2 (San Diego, CA, USA).

## Results

Twenty-four Araguaia River dolphins were successfully captured and sampled for physical and health assessment, comprising 10 individuals from the Cantão State Park and 14 from Luiz Alves area. Of these, nine individuals were males and 15 females. Most of them were classified as immature (n =  13) and, within those, three were calves and ten juveniles (Table 1).

**Table 1:** Age class, sex and body length (minimum and maximum) in centimeters (cm) of Araguaia River dolphins (n =  24) captured and released at Cantão State Park, Tocantins State, and Luiz Alves, Goiás State, Brazil, in September 2022.

Age class	Sex	n	Min length (cm)	Max length (cm)
Calf	M	2	133	157
	F	1	152	152
Juvenile	M	5	157	185
	F	5	155	180
Adult	M	2	208	230
	F	9	179	210

M=male; F=female

### Body morphology and measurements

**Fig 2:**
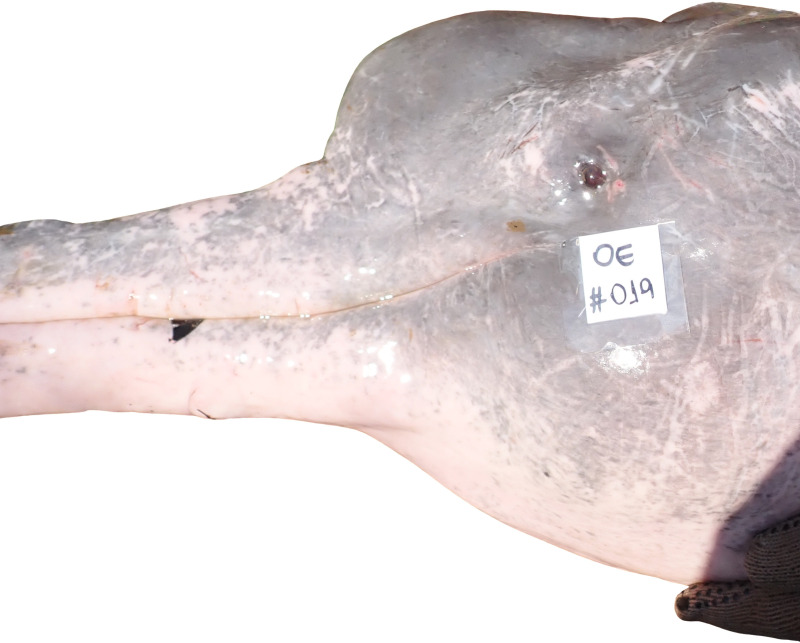
Scars from intraspeciﬁc tooth rakes were observed in the head of an adult male from Luiz Alves, Goiás, Brazil, in September 2022.

The body coloration of most female and male juveniles was entirely or predominantly gray with light pink on the ventral side. Adult females presented a medium gray body color with light pink patches at the ventral side and sometimes on the ventral side of the pectoral fluke. The body of adult males tended to present a lighter gray at the dorsal portion of the body with light pink on the sides and ventral sides. Sparse light pink was also present at the dorsal portion of the body. Scars from intraspeciﬁc tooth rakes were observed in the body of adult males, as also described for the Amazon river dolphin (Fig 2) [34].

Only two adult males were sampled, preventing a comparison of body size between adult males and females. The two adult males measured 208 and 230 cm, while the body length of adult females varied from 179 to 210 cm. Immature males and females also did not present any significant difference in body size. Body measurements of adults from Cantão and Luiz Alves did not differ, and the same occurred for juvenile dolphins from both areas.

As expected, body measurements from juveniles and adults differed in most aspects and are described in Table 2. The only difference between age classes of the same sex was the MG and W:BL, both higher in adult females (112 cm and 0.44) than immature females (90 cm and 0.31) (*P* <  0.0001, and *P* <  0.002, respectively).

**Table 2:** Body measurements of 24 Araguaia River dolphins captured and released at Cantão State Park, Tocantins State, and Luiz Alves, Goiás State, Brazil, in September 2022.

	n	Weight (kg)					Body length (cm)					Max girth (cm)					MG:BL					W:BL				
		Min	Max	Median	Mean	SD	Min	Max	Median	Mean	SD	Min	Max	Median	Mean	SD	Min	Max	Median	Mean	SD	Min	Max	Median	Mean	SD
Immature	13	42	73	54	57^*^	10	133	185	169	165^*^	14	80	102	88	89^*^	7	0.48	0.60	0.54	0.54	0.03	0.28	0.40	0.32	0.33^*^	0.04
Adults	11	78	108	88	89^*^	11	179	230	203	202^*^	13	102	124	112	114^*^	8	0.50	0.62	0.58	0.57	0.03	0.41	0.52	0.43	0.45^*^	0.04
Adult male	2	ND	ND	ND	108^a^	ND	208	230	219	219	16	122	124	123	123	1	0.5	0.62	0.58	0.57	0.04	ND	ND	ND	0.52 ^a^	ND
Adult female	9	78	99	85	86	8	179	210	199	198	9	102	124	111	112^**^	8	0.54	0.59	0.56	0.56	0.03	0.41	0.47	0.43	0.43^**^	0.02
Male immature	7	51	73	55	58	9	133	185	170	164	17	80	98	88	88	7	0.48	0.60	0.53	0.54	0.04	0.31	0.39	0.32	0.33	0.03
Female immature	6	42	73	53	55	13	152	180	166	165	11	83	102	89	90^**^	6	0.51	0.58	0.55	0.55	0.03	0.28	0.40	0.31	0.33^**^	0.05

MG:BL, relation of the maximum girth (MG) to the body length (BL); W:BL, relation of the body weight (W) to body the length (BL).

^a^Weight information available for one male

Significant difference with

*  *P* <  0.0001;

** *P* <  0.002

### Physical and health assessment

#### Physical parameters.

Despite being captured in two different geographical areas, no statistical difference was found in the total and handling time (mean =  90 min and 15 min, respectively for Cantão; and mean =  78 min and 15 min for Luiz Alves, respectively) (*P* >  0.05). Also, the stress level, cardiac rate, respiratory frequency, and oral temperature of dolphins from the two areas did not differ (*P* >  0.05). Comparison between males and females and/or between adults and immature individuals did not demonstrate statistical differences (*P* >  0.05) (Table 3).

**Table 3:** Handling time (in minutes), stress level, cardiac rate (CR), respiratory frequency (RF), and oral temperature of immature and mature Araguaia River dolphins (n =  24) captured and released in Cantão State Park, Tocantins, and Luiz Alves, Goiás, Brazil, in September 2022.

Immature (n = 13)						Adults (n = 11)					*P*
	Min	Max	Median	Mean	SD	Min	Max	Median	Mean	SD	
Handling time (min)	11	21	14	15	2.9	12	24	15	17	4.0	0.29
Total time (min)	19	149	79	80	37.4	26	179	65	87	48.3	0.91
Stress	2	4	3	3	0.8	3	4	4	4	0.5	0.31
CR (beats/min)	80	122	100	99	11.7	80	112	89	91	10.5	0.09
RF (breaths/min)	2	12	6	7	2.4	2	9	6	6	2.4	0.74
Oral Temp (˚C)	33	37	34	35	1.3	34	37	35	35	1.2	0.36

#### Hematology.

The hematological variables of males and females and/or dolphins from different areas were not significantly different. On the other hand, juvenile dolphins presented higher WBC and lymphocytes than adults, as described in Table 4. Positive correlations of the blood constituents were found between RBC and HCT, WBC and lymphocytes, and among hematimetric indices (S1 and S2 Tables). No correlation was found between any hematological parameter and cardiac rate, respiratory frequency, oral temperature, or stress level (*P* >  0.05). Most mean hematological values in the Araguaia River dolphin fell beyond the reference intervals established for the Amazon river dolphins*.* However, the minimum and maximum ranges are within the reference values described for the Amazon river dolphin (Table 4).

**Table 4:** Hematological values of immature and mature Araguaia River dolphins (n =  24) captured and released at Cantão State Park, Tocantins and Luiz Alves, Goiás, Brazil, in September 2022; and 90% lower and higher confidence intervals (reference values) of subadults and adults of Amazon river dolphin [18].

	Immature (n = 13)						Adults (n = 11)							Amazon river dolphin	
	Unit	Min	Max	Median	Mean	SD	Min	Max	Median	Mean	SD	P	Dist	90% Lower threshold	90% Higher threshold
HCT	L/L	0.38	0.48	0.42	0.43	0.03	0.39	0.46	0.44	0.43	0.03	0.93	G	0.39	0.4
RBC count	10^12^/L	3.41	4.74	3.72	3.9	0.45	3.27	4.29	3.58	3.71	0.36	0.67	NG	3.8	4.02
Hemoglobin	g/L	123.1	147.5	137.8	136.6	8.5	123.8	147.1	136.9	136.6	7	0.98	G	149.5	162.4
MCV	fL	94.94	121.13	110.34	111.05	6.90	103.53	128.44	116.88	115.80	7.78	0.14	G	101	107
MCH	fmol	1.74	2.53	2.27	2.18	0.26	1.99	2.59	2.25	2.31	0.19	0.21	G	ND	ND
MCHC	g/L	256.5	347.5	325.3	319	24.8	298.9	344.9	319.6	321.7	13.6	0.17	NG	379.4	394.1
WBC count	10^9^/L	5.8	12.45	8.9	**9.29**	2.12	4.9	12.95	6.45	**7.02**	2.24	**0.02**	G	15.81	17.44
Monocyte	%	2	8	4	4.54	2.11	1	7	3	3.45	2.3	0.24	G	2.47	3.24
Monocyte	10^9^/L	1.74	6.19	3.23	3.68	1.69	0.58	5.7	1.53	2.45	1.83	0.13	G	0.35	1.04
Lymphocyte	%	26	54	39	39.85	8.89	23	47	33	33.3	6.96	0.07	G	33.41	36.75
Lymphocyte	10^9^/L	2.21	5.35	3.89	**3.48**	1.1	1.47	4.92	2.07	**2.38**	1.04	**0.002**	NG	5.68	6.91
Basophil	%	0	1	0	**0.08**	0.28	0	1	0	**0.18**	0.4	**0.001**	NG	ND	ND
Basophil	10^9^/L	0.00	0.82	0.00	0.07	0.02	0.00	0.64	0.00	0.1	0.023	0.94	NG	ND	ND
Eosinophil	%	2	8	5	5	2.27	2	10	7	6.5	2.64	0.10	NG	11.86	14.27
Eosinophil	10^9^/L	1.44	8.71	4.76	4.64	0.24	1.29	6.6	4.75	4.43	0.17	0.82	G	2.04	2.79
Neutrophil	%	35	66	52	51.5	8.4	48	65	54.5	55.3	5.6	0.24	G	46.16	49.65
Neutrophil	10^9^/L	2.88	6.36	4.42	4.57	1.12	2.5	6.73	3.42	4.03	1.29	0.29	G	7.64	8.91
Band neut	%	0	2	0	0.38	0.65	0	2	0	0.55	0.69	0.75	NG	0.83	1.3
Band neut	10^9^/L	0.00	1.19	0.00	0.22	0.41	0.00	1.29	0.00	0.36	0.47	0.94	NG	0.14	0.24
NLR		0.70	2.54	1.37	1.45	0.58	1.02	2.83	1.65	1.76	0.54	0.23	G	ND	ND

HCT, hematocrit; RBC, red blood cell; MCV, mean corpuscular volume; MCH, mean corpuscular hemoglobin; MCHC, mean corpuscular hemoglobin concentration; WBC, white blood cell; Band neut, band neutrophils; NLR, neutrophil-lymphocyte ratio. Dist, distribution; G =  Guassian, NG =  Non-Guassian, ND =  Not Determined

#### Serum Chemistry.

The serum chemistry of males and females did not differ. However, differences were detected between age classes. Uric acid was higher in adult individuals, while the values for serum of ALP, CK, and phosphate were higher in juveniles (Table 5). Unlike the hematological values, variation was observed in dolphins from the two distinct areas. Dolphins from Cantão had higher serum AST and urea/creatinine and lower GGT, total bilirubin, and creatinine than dolphins from Luiz Alves (Table 6). A statistically significant correlation was found between the total handling time and serum glucose (r_S_ =  0.44, *P* =  0.03). Cardiac rate correlated with serum ALP (r_S_ =  0.55, *P* =  0.008), CK (r_S_ =  0.57, *P* =  0.007), uric acid (r_S_ =  0.47, *P* =  0.03) and phosphate (r_S_ =  0.43, *P* =  0.04). Other correlations between serum chemistry analytes are presented in the S2 Table. Additionally, the mean serum chemistry values of the Araguaia River dolphin were within reference intervals established for 107 healthy Amazon river dolphins [21].

**Table 5:** Serum chemistry values of immature and mature Araguaia River dolphins (n =  24) captured and released at Cantão State Park, Tocantins, and Luiz Alves, Goiás, Brazil, in September 2022; and 90% lower and higher confidence intervals (reference values) of calves, juveniles and adults of Amazon river dolphin [21].

	Unit	Immature (n = 13)					Adults (n = 11)					*P*	Dist.	Amazon river dolphin	
		Min	Max	Median	Mean	SD	Min	Max	Median	Mean	SD			90% Lower threshold	90% Higher threshold
Albumin	g/dl	2.23	2.67	2.40	2.46	0.14	2.38	2.76	2.58	2.57	0.13	0.053	G	1.89	5.80
Total protein	g/dl	7.34	9.07	8.08	8.19	0.51	7.83	9.10	8.40	8.46	0.39	0.182	G	5.89	14.63
Globulin	g/dl	5.37	6.68	5.69	5.86	0.47	5.25	6.37	5.96	5.88	0.35	0.879	G	4.60	9.47
Album/Glob		0.38	0.60	0.45	0.46	0.06	0.39	0.49	0.44	0.44	0.03	0.245	NG	ND	ND
Calcium	mmol/L	2.37	2.64	2.52	2.51	0.07	2.35	2.64	2.46	2.48	0.1	0.489	G	2.44	5.58
Glucose	mmol/L	4.66	9.88	7.27	7.28	1.53	6.16	1.82	7.83	8.04	1.75	0.269	G	4.96	19.9
Cholesterol	mmol/L	5.12	8.38	6.1	6.38	1.6	4.73	8.02	6.96	6.9	0.86	0.241	G	3.85	15.1
Triglycerides	mg/dL	0.69	1.55	0.96	0.99	0.26	0.51	1.58	0.96	0.98	0.35	0.883	G	0.43	2.85
ALP	µkat/L	1.27	4.53	3.28	**3.1**	0.89	0.75	2.46	1.23	**1.48**	0.6	**<0.0001**	G	0.65	2.96
ALT	µkat/L	0.06	0.17	0.1	0.1	0.03	0.07	0.21	0.13	0.13	0.05	0.079	G	0.05	6.6
Amylase	µkat/L	0.58	1.58	0.95	1.03	0.35	0.65	1.73	1.03	1.12	0.36	0.544		2.52	19.87
Lipase	µkat/L	0.35	0.67	0.38	0.47	0.17	0.15	2.85	0.28	0.89	1.31	0.609	G	0.12	0.41
AST	µkat/L	1.29	1.79	1.59	1.58	0.16	1.23	1.84	1.59	1.52	0.19	0.471	G	1.28	6.49
CK	µkat/L	2.32	5.43	3.57	**3.73**	0.97	0.98	4.55	2.31	**2.53**	1.12	**0.011**	G	0.28	7.41
GGT	µkat/L	0.29	0.46	0.38	0.38	0.05	0.29	0.4	0.39	0.35	0.03	0.093	G	0.19	0.59
Total Bili.	µmol/L	1.88	5.99	3.42	3.76	1.20	2.22	5.64	3.59	3.76	1.03	0.802	G	3.42	39.672
Direct Bili.	µmol/L	0.17	0.51	0.34	0.34	0.17	0.17	0.68	0.34	0.51	0.17	0.328	NG	1.71	17.955
Indirect Bili.	µmol/L	1.54	5.81	3.08	3.25	1.20	1.88	4.96	3.08	3.25	1.03	0.906	G	1.71	21.20
Creatinine	µmol/L	92.84	237.85	171.53	169.77	54.82	129.98	246.69	174.19	186.57	43.33	0.439	G	121.14	242.27
Urea	mmol/L	15.37	41.22	28.52	29.02	7.16	15.17	50.50	38.65	36.52	11.72	0.067	G	18.60	70.87
Urea/Creat		16.36	96.21	53.00	49.12	24.69	15.50	86.30	54.95	53.11	23.68	0.692	G	ND	ND
Uric acid	µmol/L	273.61	374.72	299.18	**306.92**	30.33	297.40	381.27	350.93	**349.15**	26.77	**0.003**	G	163.57	365.21
Phosphate	mmol/L	1.68	2.90	2.38	**2.32**	0.34	1.75	2.40	2.05	**2.07**	0.23	**0.047**	G	1.14	2.85

ALP, alkaline phosphatase; ALT, alanine aminotransferase; AST, aspartate transaminase; CK, creatine kinase; GGT, gamma-glutamyl transferase; Dist, distribution; Bili, bilirubin; Urea/Create, urea creatinine relation; G, Gaussian; NG, Non-Gaussian; ND, Not determined.

**Table 6:** Serum chemistry values of Araguaia River dolphins (n =  24) captured and released at Cantão State Park, Tocantins state, and Luiz Alves, Goiás state, Brazil, in September 2022.

	Unit	Cantão (n = 10)					Luiz Alves (n = 14)					*P*	Dist
		Min	Max	Median	Mean	SD	Min	Max	Median	Mean	SD		
Albumin	g/dl	2.23	2.76	2.42	2.45	0.15	2.29	2.73	2.56	2.55	0.13	0.103	G
Total protein	g/dl	7.89	8.97	8.39	8.40	0.38	7.34	9.10	8.19	8.27	0.52	0.530	G
Globulin	g/dl	5.37	6.68	6.03	5.98	0.39	5.25	6.53	5.70	5.78	0.41	0.272	G
Album/Glob		0.39	0.60	0.44	0.45	0.06	0.38	0.57	0.45	0.45	0.05	0.978	NG
Calcium	mmol/L	2.39	2.55	2.48	2.48	0.06	2.35	2.65	2.54	2.52	0.10	0.303	G
Glucose	mmol/L	5.94	10.73	8.17	8.20	1.75	4.62	10.51	7.04	7.10	1.43	0.106	G
Cholesterol	mmol/L	5.94	9.35	7.93	7.72	1.28	5.48	9.72	7.47	7.61	1.22	0.836	G
Triglycerides	mmol/L	0.49	1.49	0.98	0.98	0.31	0.66	1.54	0.88	0.95	0.28	0.811	G
ALP	µkat/L	1.15	3.60	2.33	2.30	0.97	0.77	4.62	2.28	2.42	1.27	0.813	G
ALT	µkat/L	0.06	0.21	0.12	0.13	0.05	0.06	0.18	0.11	0.12	0.03	0.552	G
Amylase	µkat/L	0.60	1.62	1.11	1.06	0.35	0.66	1.77	0.94	1.12	0.37	0.697	NG
Lipase	µkat/L	0.15	0.39	0.29	0.28	0.11	0.20	0.68	0.36	0.41	0.24	0.186	G
AST	µkat/L	1.49	1.89	1.68	**1.69**	0.11	1.26	1.83	1.53	**1.53**	0.19	**0.049**	G
CK	µkat/L	1.80	5.27	3.64	3.61	1.15	1.00	5.54	2.69	2.97	1.22	0.225	G
GGT	µkat/L	0.29	0.40	0.36	**0.35**	0.04	0.34	0.46	0.39	**0.39**	0.04	**0.029**	G
Total Bili.	µmol/L	1.88	5.13	3.08	**3.08**	0.86	2.91	5.99	3.93	**4.10**	1.03	**0.022**	G
Direct Bili.	µmol/L	0.17	0.51	0.34	0.34	0.00	0.17	0.68	0.51	0.51	0.17	0.122	NG
Indirect Bili.	µmol/L	1.54	4.62	2.74	2.74	0.86	1.88	5.81	3.42	3.59	1.20	0.066	G
Creatinine	µmol/L	92.84	227.24	136.17	**143.24**	41.56	132.63	246.69	218.40	**202.48**	39.79	**0.002**	G
Urea	mmol/L	26.16	49.71	35.68	36.64	8.14	15.29	50.93	30.64	29.93	10.75	0.111	G
Urea/Creat		30.20	96.21	69.34	**67.02**	20.17	15.50	74.06	38.58	**39.48**	19.48	**0.003**	G
Uric acid	µmol/L	273.61	346.17	303.35	309.89	27.36	279.56	381.27	339.04	333.68	37.47	0.115	G
Phosphate	mmol/L	1.63	2.82	2.24	2.15	0.33	1.69	2.62	2.09	2.12	0.29	0.824	G

ALP, alkaline phosphatase; ALT, alanine aminotransferase; AST, aspartate transaminase; CK, creatine kinase; GGT, gamma-glutamyl transferase; Dist, distribution; Bili, bilirubin; Urea/Create, urea creatinine relation; G, Gaussian; NG, Non-Gaussian; ND, Not determined.

#### Endocrine assessment.

All tested hormones were detectable in the serum of the Araguaia River dolphins. The mean intra-assay coefficient of variation was 5.04% for testosterone, 5.82% for cortisol, 5.1% for progesterone, and 12.93% for estradiol. All assays passed parallelism tests in serial dilutions of 1:1 to 1:8 for cortisol, and 1:1 to 1:16 for testosterone, progesterone, and estradiol. The slopes were >  0.05 for all hormones: Testosterone: F_1,10_ =  0.95, *P* =  0.35; Cortisol: F_1,5_ =  2.56, *P* =  0.17; Progesterone: F_1,8_ =  3.03, *P* =  0.12; Estradiol: F_1,7_ =  0.06, *P* =  0.81 (S1 Fig). Accuracy was r^2^ >  0.95 and slope within the range of 0.7 – 1.3 for testosterone, progesterone, and cortisol. The slope of the estradiol was 0.49 (S1 Fig).

Reproductive hormones did not differ between the individuals from the sampling areas. However, as expected, some differences were found between the sexes (Table 7), and correlations were found between the serum hormone levels and body measurements. No statistical analyses were performed between male and female juveniles or between male and female adults given the low number of individuals per category. Descriptive data for those are presented in Table 7.

**Table 7:** Serum steroid hormones of 24 Araguaia River dolphins captured and released at Cantão State Park, Tocantins, and Luiz Alves, Goiás, Brazil, in September 2022. Statistical analysis (Mann-Whitney test) was performed only between males and females.

	n	Testosterone					Progesterone					Estradiol					Cortisol				
		Min	Max	Median	Mean	SD	Min	Max	Median	Mean	SD	Min	Max	Median	Mean	SD	Min	Max	Median	Mean	SD
Male	9	0.24	7.58	0.54	**1.70** ^*^	2.55	0.17	0.76	0.39	0.46	0.25	22.96	72.92	50.17	48.50	17.42	14.85	40.60	27.67	26.65	8.57
Female	15	0.05	2.35	0.11	**0.53** ^*^	0.73	0.01	3.44	0.32	1.04	1.18	16.25	107.80	36.45	44.17	25.70	8.94	48.11	27.96	27.18	10.22
Male juvenile	7	0.24	0.89	0.40	0.49	0.24	0.17	0.76	0.39	0.46	0.26	22.96	72.92	50.17	50.24	19.77	17.94	40.60	24.79	27.29	9.16
Female juvenile	6	0.05	0.23	0.07	0.09	0.07	0.01	0.61	0.17	0.24	0.21	26.19	40.05	34.39	33.45	5.70	8.94	34.88	28.35	24.65	9.76
Adult male	2	3.06	7.58	5.32	5.32	3.20	0.20	0.70	0.45	0.45	0.35	34.06	54.28	44.17	44.17	14.30	30.54	31.34	30.94	30.94	0.57
Adult female	9	0.05	2.35	0.57	0.82	0.82	0.13	3.44	1.67	1.57	1.27	16.25	107.80	43.40	52.21	32.12	14.45	48.11	27.96	28.86	10.73

* *P* = 0.04.

**Fig 3:**
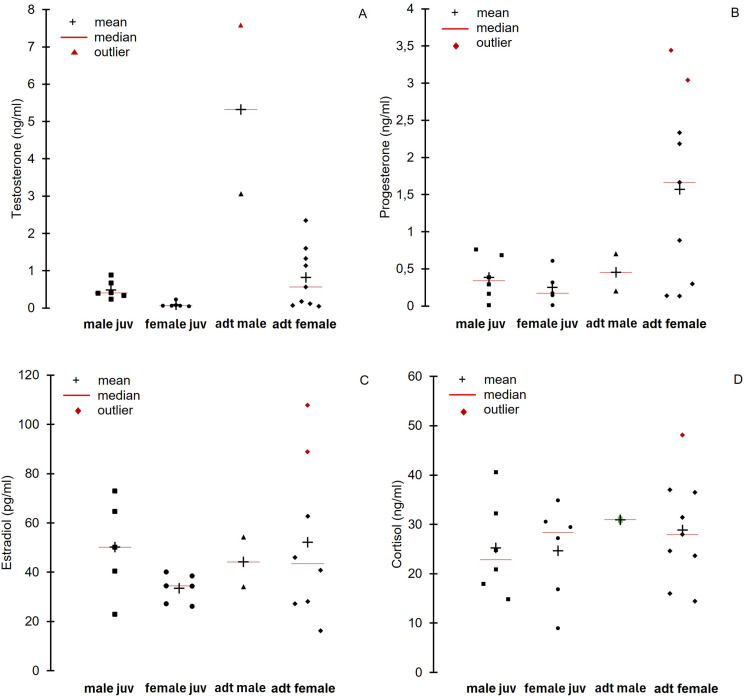
Mean, median, and outlier values of serum steroid hormones of 24 Araguaia River dolphins captured and released at Cantão State Park, Tocantins state, and Luiz Alves, Goiás state, Brazil, in September 2022.

Male Araguaia River dolphins presented higher mean serum testosterone than females (Table 7). The two highest serum testosterone values ( > 3.06 ng/ml) were detected for the two adult males sampled, while the highest serum testosterone value for females was 2.35 ng/ml (Fig 3A). A positive correlation was observed between serum testosterone and body length of all individuals (r_S_ =  0.54, *P* =  0.009), body weight (r_S_ =  0.61, *P* =  0.01), and the relation W: BL (r_S_ =  0.64, *P* =  0.01). The W: BL correlation is no longer statistically significant for males when individuals are separated by sex but still significant for females: males (r_S_ =  0.60, *P* =  0.24) and females (r_S_ =  0.70, *P* =  0.01).

Mean serum progesterone was higher in adults (1.37 ng/ml) than in juveniles (0.25 ng/ml) (*P* =  0.04) and did not differ between males and females (Table 7). However, only adult females had the highest progesterone outlier values (Fig 3B). A positive correlation was found between serum progesterone and weight (r_S_ =  0.59, *P* =  0.02) and W: BL (r_S_ =  0.61, *P* =  0.01) in all animals and between body length and progesterone in females (r_S_ =  0.52, *P* <  0.0001). Serum estradiol outlier value was also detected in one adult female (Fig 3C); however, no differences were found between males and females (Table 6). No correlation was found between any of the reproductive hormones (*P* >  0.05).

Mean serum cortisol did not differ between dolphins from Cantão (mean =  29.51 ng/ml) and Luiz Alves areas (mean =  25.05 ng/ml) (*P* =  0.27) or between males and females (Table 7; Fig 3D). Adults and juveniles also did not differ in serum cortisol (29.24 ng/ml and 24.94 ng/ml, respectively) (*P* =  0.29). No correlation between body measurements or physical parameters and cortisol was found. Serum cortisol negatively correlated with RBC (r_S_ =  -0.485, *P* =  0.02), and the percentage of neutrophils (NEU (%)) (r_S_ =  -0.455, *P* =  0.03). A positive correlation was observed between cortisol levels and serum glucose (r_S_ =  0.440, *P* =  0.04). No correlation was observed between cortisol and the reproductive hormones.

## Discussion

This study provides the first complete assessment of the physical characteristics and health of the Araguaia River dolphin. Most of the dolphins captured in the present study comprised mother-calf pairs and/or juveniles. This can be explained by the most frequent occupancy of these life history groups into areas with calm waters similar to small river channels and bays where the nets were deployed. Also, younger animals are slower to escape the net, and mothers tend to stay close to their calves, a behavior also observed in the Bolivian river dolphin (*I. boliviensis*) [38]. The preliminary information on the external morphology and body measurements from individuals inhabiting these two Araguaia River areas, more than 500 km apart, did not show any apparent differences. This is congruent with other investigations regarding potential intra or interspecific differences, such as molecular and skull morphology characters from dead individuals sampled along different locations of the Araguaia River [39,40].

The body coloration of immature animals was dark grey, while adult individuals were predominantly medium gray with light pink on the ventral side. Adult males tended to have more light pink areas in the body than females and immature individuals. A similar color pattern was observed for the Araguaia River dolphins from the Tocantins River in Pará state, Brazil [41]. The total body length of the Araguaia dolphins from the present study varied from 133 to 230 cm, with the adult males reaching 230 cm and the females 210 cm. A previous study reported the largest male (n =  9) being 236 cm long and the largest female (n =  12) 201 cm long from areas below and above Tucuruí Dam in the Tocantins River, Brazil [41]. The interval observed in the present study is also inside the body lengths found for its counterpart dolphin species, the Amazon river dolphin and the Bolivian river dolphin [38,42]. Within age classes, most of the body length of adult males and females was also in the interval found for Amazon river dolphins [22,43]. Juvenile females (155 – 180 cm) and males (157-185 cm) presented a similar total body length to juveniles of Amazon river dolphins (females 151-166 cm, and males 169-189 cm) [18]. Preliminary studies on the skull morphometrics of the genus *Inia* could not detect any significant differences between *I. geoffrensis* and *I. araguaiaensis* [40]. However, the authors state that the failure to identify significant differences between these two *Inia* lineages does not necessarily imply they are not different species, as the absence of morphometric differentiation may be present within reproductively isolated species in cetaceans [39,43,44].

Preliminary information on the species’ physiology and health is presented. It should be noted that a bias on these aspects might be present, as they are free-ranging dolphins being caught and released for research purposes. It has been shown that the time of capturing and handling wild dolphins can have some influence on some of their physical and blood parameters [16,45]. For instance, the empirical observation of four different stress states positively correlated with the cardiac rate and respiratory frequency in the Amazon river dolphin [22]. The values of these two parameters of Araguaia River dolphins were within the interval found for the Amazon river dolphins and did not correlate with any of the blood values or the capture and handling time. However, the low number of dolphins analyzed in the present study may have contributed to the lack of correlation among elements that could potentially indicate distress. The oral temperature (OT) varied from 33 to 37 ˚C and was described for the first time for a cetacean species. Other approaches have been used to measure the body temperature of dolphins such as rectal probes, and infrared thermography [46–48]. The mean OT did not vary between immature and adult individuals and the narrow range of the minimum and maximum values demonstrates its potential as a proxy to assess the expected body temperature in river dolphins, assuming that all individuals in the present study were considered healthy. Since the OT is not a direct measure of the core body temperature, some variation may occur as a result of thermoregulation by peripheral vasodilation or vasoconstriction according to the water or atmosphere temperature [49]. However, the range limits of OT should be expected to be in between known physiological values.

Most of the mean hematological parameters for the Araguaia River dolphins were outside the reference intervals previously described for the Amazon river dolphins inhabiting the Mamirauá Sustainable Development Reserve (RDSM) and surroundings, but inside its minimum and maximum ranges [18]. Exceptions to this are the hemoglobin and the leucocyte’s total and relative counts, which were consistently lower and outside of all values described for the Amazon river dolphin [22]. The reference values of hemoglobin described for Amazon river dolphins were 149 - 162 g/L, and its values varied from 122 g/dL to 390 g/dL, while the hemoglobin concentration for Araguaia River dolphins varied from 123 – 147.8 g/dL. Since the hematological techniques employed were the same for the two species, we may infer that this likely represents a result of the environmental conditions in their physiology [22,35]. It is known that dolphins’ red blood parameters may be increased as physiological plasticity to extend aerobic dive limits in terms of water depth or the need for extended dives according to the environment [50,51]. The Araguaia River has a mean water depth of 4.3 m at mean annual discharge and of the order of 1.5 – 2.0 m for the dry season [32,52], while the mean water depth in lakes and channels of the Mamirauá area is 6.2 – 8.5 m and 7.9 – 10.6 m, respectively [53]. Offshore ecotypes of bottlenose dolphins have higher hemoglobin levels, packed cell volumes, and red blood cell counts than coastal specimens, which gives them a higher need for oxygen supply during deeper dives [54]. RBCs, HCT, and MCV were higher in the Yangtze Finless porpoise (*Neophocaena asiaeorientalis*) inhabiting an area with heavier vessel traffic (5.3 x 10^12^/L; 0.47 L/L; and 89.9 fL, respectively) than in a protected area (4.9 x 10^12^/L; 0.43 L/L; and 86.0 fL, respectively), probably because of extended dive periods to avoid the boats [15]. The lower Hb levels of the Araguaia dolphins from the present study may be related to the predominantly shallower environment of the Araguaia River compared to the rivers in the central Amazon.

Immunogenic stimulation may reflect the number of WBCs on a determined species, especially within age classes, parasitic load, and diseases [22,55]. WBCs were higher in Yangtze Finless porpoises living in an area with intense underwater noise and heavy vessel traffic compared to porpoises living in a protected area [15]. The WBC of dolphins from the protected area of Cantão State Park did not differ from dolphins sampled in Luiz Alves, despite the more intense human activity in the second. Indeed, no other hematological parameters differed between these two areas or between males and females. However, immature individuals showed higher WBC and lymphocytes than adults in both regions. The number of leukocytes is expected to be higher in most still-developing mammals, including cetaceans [18,56,57]. Some expected correlations were found among the blood constituents since one variable directly influences the value of another. For instance, the RBC and Hb values are positively correlated to the HCT, while the WBC increase was directly influenced by the increased number of lymphocytes and neutrophils. Similar correlations were found in other species as bottlenose dolphins (*Tursiops truncatus*), Amazon River dolphins, belugas (*Delphinapterus leucas*), Dall’s porpoises (*Phocoenoides dalli*), and the Pacific white-sided dolphins (*Lagenorhynchus obliquidens*), among others [18,58,59].

The serum chemistry values of the Araguaia River dolphins were similar to those of Amazon river dolphins, with all values falling in the reference interval established for 107 healthy individuals [21]. Five of the 23 serum chemistry investigated diverged between the localities of Cantão and Luiz Alves (Table 6). Given the low number of sampled individuals, the differences in serum analytes should be interpreted with caution. Mean serum AST activity was higher in the dolphins from the Cantão area; however, it is within the minimum and maximum range found for dolphins from Luiz Alves. Higher serum AST activity is usually associated with liver disorders, heart diseases, or skeletal muscle damage after strenuous exercise or restraint [60]. CK is also a valuable bioindicator for the detection of muscle damage after wildlife capture [61]. Although no statistical difference was observed in the CK mean between dolphins of the locations, the individuals from Cantão had a higher mean for the activity of this enzyme. This may indicate a more pronounced muscle trauma during the capture and restraint of the dolphins in this area.

The higher serum creatinine and lower urea/creatinine in the dolphins from Luiz Alves may result from intrinsic physiological variations related to adaptative plasticity between the two populations or, less likely, indicate abnormal renal function. Abnormally increased serum creatinine is often associated with reduced kidney function [62]. Different prey types and/or consumption may also alter the serum creatinine as it may be elevated after a protein-rich consumption [63]. Muscle mass may also influence serum creatinine levels since creatinine is slowly broken down at a rate directly proportional to muscle mass [64]. A positive correlation between body mass and serum creatinine was found for different species of free-ranging cetaceans dolphins, including the bottlenose dolphin, beluga, and the Amazon River dolphins [21,65,66]. Although not statistically different, the total mean body mass of dolphins from Luiz Alves (76 kg) was more elevated than that of dolphins from Cantão (65.5 kg). Future investigation should be carried out to clarify if these differences are related to distinct prey types, total body mass, or the occurrence of subclinical diseases in the Araguaia River dolphins.

Serum means for total bilirubin, and the activity of GGT were higher in the dolphins sampled in Luiz Alves. Usually, these elements are more likely to be related to a health imbalance/disruption than a physiological adjustment to environmental conditions as diet differences. In aquatic mammals, elevated bilirubin levels are usually associated with obstructive intrahepatic disease or increased RBC lysis [60,67]. GGT may be elevated in obstructive liver disease and cholestasis in dolphins [60]. Although no clinical signs of illness were detected during the health assessment, subclinical diseases may be present in the population. Thus, follow-up studies related to investigating infectious and parasitic diseases are recommended.

Reproductive hormones followed the general tendency that has been reported in other mammals, with the highest values of serum testosterone detected in adult males and the highest values for serum progesterone and estradiol in adult females [68]. No statistical differences were detected between the serum hormone concentrations of dolphins from the two localities. Since there is no information regarding the species’ basic reproductive biology and physiology, the hormone data represents a very preliminary step to understanding them. Also, the potential plasticity in hormone levels within and among individuals over time, given seasonality, should be considered.

Several correlations to the body measurements were detected, demonstrating a preliminary variation of the reproductive hormones accompanying the body development as the individuals reach sexual maturity in both localities. The mean serum testosterone levels of the two adult males of Araguaia River dolphins (5.32 ng/ml) were analogous to those found in the adult males of Amazon river dolphins ( > 5 ng/ml) and Yangtze ﬁnless porpoise males during the reproductive period (5.02 ±  3.20 ng/ml) [69,70]. As expected, male dolphins in the present study presented higher testosterone serum levels than females possibly as a result of testicular activity [70,71]. Adult females also had a higher mean for testosterone than immature females. Testosterone is an important hormone in the female reproductive biology of mammals and tends to be elevated during gestation in dolphins [72,73]. Serum testosterone tends to increase during pregnancy in female Amazon river dolphins [70].

A positive correlation was observed between serum testosterone and body length of all individuals (r_S_ =  0.54, *P* =  0.009), body weight (r_S_ =  0.61, *P* =  0.01), and the relation W: BL (r_S_ =  0.64, *P* =  0.01). Besides the expected higher reproductive hormones after the onset of puberty, elevated testosterone levels have been reported to increase in pregnant cetaceans, including humpback whales (*Megaptera novaeangliae*), belugas, killer whales, bottlenose dolphins, and Amazon river dolphins [70,74–76]. The higher W: BL in females than males and a positive correlation between testosterone and W: BL may indicate an increased belly girth in a potential pregnancy. Some adult females showed a protruding belly, although ultrasonography in the field could not confirm the pregnancy.

The serum progesterone of female Araguaia River dolphins varied from 0.01 – 3.44 ng/ml. This represents a substantially lower range for the progesterone found for pregnant free-ranging Yangtze ﬁnless porpoise (13.2 to 112.4 ng/mL) and slightly higher for non-pregnant females (under 1.0 ng/mL). A 4.5 to 21.6 ng/ml variation was observed in 58 free-ranging pregnant females of Amazon river dolphins [70]. The progesterone levels indicating a potential pregnancy are highly variable among cetaceans, with elevation of progesterone occurring post-ovulation [76]. It usually remains elevated until luteolysis for most species but may gradually decline, as demonstrated in killer whales (*Orcinus orca*) [77,78]. A positive correlation was found between progesterone and weight (r_S_ =  0.59, *P* =  0.02) and W: BL (r_S_ =  0.61, *P* =  0.01) in all individuals and between body length and progesterone in females (r_S_ =  0.52, *P* <  0.0001). Since there is no available information on the species’ reproductive biology, further studies are warranted, preferably with all-year-round sampling, to better interpret the role of progesterone in the pregnancy of Araguaia River dolphins. Estradiol is mainly synthesized in the ovarian follicles, and it is usually higher in females than males [78,79]. However, estradiol in both male and female cetaceans can be relatively low and overlap outside the reproductive period [69]. Although no statistical difference was observed between males and females, only adult females presented outliers with the highest values.

Cortisol is commonly used as a biomarker to detect stressful events such as heavy boat traffic, underwater noise, and pollution, among other anthropogenic disturbances in cetaceans [15,23,80,81]. Araguaia River dolphins inhabiting areas with different degrees of human activities did not show differences in the serum cortisol. Also, no differences were detected in serum cortisol between sex and age classes. The range found for the Araguaia River dolphins (8.94 – 48.11 ng/ml) was broader than the values found for the Yangtze Finless porpoise (0.79 to 28.9 ng/ml) in different seasons of the year [22]. The lack of differences in the cortisol levels between dolphins from the two areas in the Araguaia River may open space for interpretations given the plasticity and the time of incorporation of this hormone into the cetacean’s different tissues commonly used for endocrine monitoring. It has been demonstrated that the measurement of cortisol in the baleen and blubber of whales can be used as a tool to monitor the past weeks to months or years in an individual’s life [82,83]. On the other hand, serum cortisol reflects the instantaneous concentration of this hormone in the blood. The acute stress suffered during the capture and restraint of the dolphins in both areas could have masked the potential long-term stress suffered by dolphins inhabiting the intense human activity area in Luiz Alves. An abnormal increase in the activity of the hypothalamic-pituitary-adrenal axis (HPA) during capture may have led to an unusually high secretion of glucocorticoids into the bloodstream. Apart from the activation of HPA, the activation of the sympathetic nervous system where the catecholamines modulate the cardiac rate, respiratory frequency, and mobilization of blood cells into the bloodstream such as WBC and neutrophils may occur in stress-inducing events [84,85].

Serum cortisol did not correlate with any of the physical parameters (e.g., cardiac rate, respiratory frequency, handling time), and as opposed to expected, the cortisol levels negatively correlated to RBC and NEU (%). Empirical observation of the “level of stress” from Amazon River dolphins from a capture-and-release program of Projeto Boto, Amazonas, Brazil, correlated with a series of physical indices like cardiac rate and respiratory frequency and with some blood parameters including WBC and neutrophils [18]. However, the serum cortisol of Araguaia River dolphins positively correlated with glucose plasma concentration, potentially demonstrating the activation of the HPA during capture, but not in a timely linear progressive increase, as it did not correlate with capture/handling time. In this sense, measuring serum cortisol may not be efficient in detecting acute stress events in wild dolphins. Mid-term and chronic stressors, comprising anthropogenic impacts, may be more effectively detected by the measurement of this hormone in other biological matrices such as blubber and skin [86–89].

Despite the urgent need for knowledge of the physical and health parameters of Araguaia River dolphins, this data set does not represent long-time monitoring and does not embrace the minimum number of individuals to calculate reference values to the species. Thus, we recommend that future studies increase the number of sampled individuals to generate more robust data and results across locations and age classes and, if possible, during different periods of the year.

## Conclusions

Preliminary data showed that most physical and healthy parameters did not vary between Araguaia River dolphins of areas with different degrees of human activity. Exceptions to this were found among some serum analyte concentrations, which may result from different prey types, intensities of muscle extenuation during capture, or subclinical diseases. Few differences occurred between Araguaia River dolphins and Amazon river dolphins regarding external body appearance and blood values. Generally, the Araguaia River dolphins tend to present greyer bodies than their counterparts inhabiting the Amazon Basin. Most hematological and serum chemistry were within the minimum and maximum values described for the Amazon River dolphins. However, some variation between the two species may reflect the physiological plasticity needed to cope with different environments. Immature and mature individuals showed differences in the blood variables related to body and immunological development. Reproductive hormones correlate with most body measurements, demonstrating a potential increase in gonadal activity as individuals reach sexual maturity. Our findings may provide preliminary, unprecedented information on the physical and health parameters of the Araguaia River dolphin. This information is essential to proper management and conservation efforts, including national and international protection laws.

## Supporting information

S1 Fig Parallelism (on the left side) and accuracy (on the right side) graphs tested with serum pooled extract for testosterone, progesterone, estradiol, and cortisol of *Inia araguaiaensis* captured and released at Cantão State Park, Tocantins state, and Luiz Alves, Goiás state, Brazil, in September 2022. Dilutions with no detectable hormone are not shown.
(JPG)

S1 Table Correlation among red blood indices of 24 Araguaia River dolphins captured and released at Cantão State Park, Tocantins, and Luiz Alves, Goiás, Brazil, in September 2022. (DOCX)

S2 Table Correlation among white blood cell indices of 24 Araguaia River dolphins captured and released at Cantão State Park, Tocantins, and Luiz Alves, Goiás, Brazil, in September 2022. (DOCX)

S3 Table Araguaia river dolphin Dataset. (XLSX)
