# Supplementary figures and images for: Comprehensive assessment of the physical and health features of the threatened Araguaian River dolphin Inia araguaiaensis
Source: PLoS One. 2025 Mar 31;20(3):e0319212. doi: 10.1371/journal.pone.0319212 (PMC11957337; doi:10.1371/journal.pone.0319212)

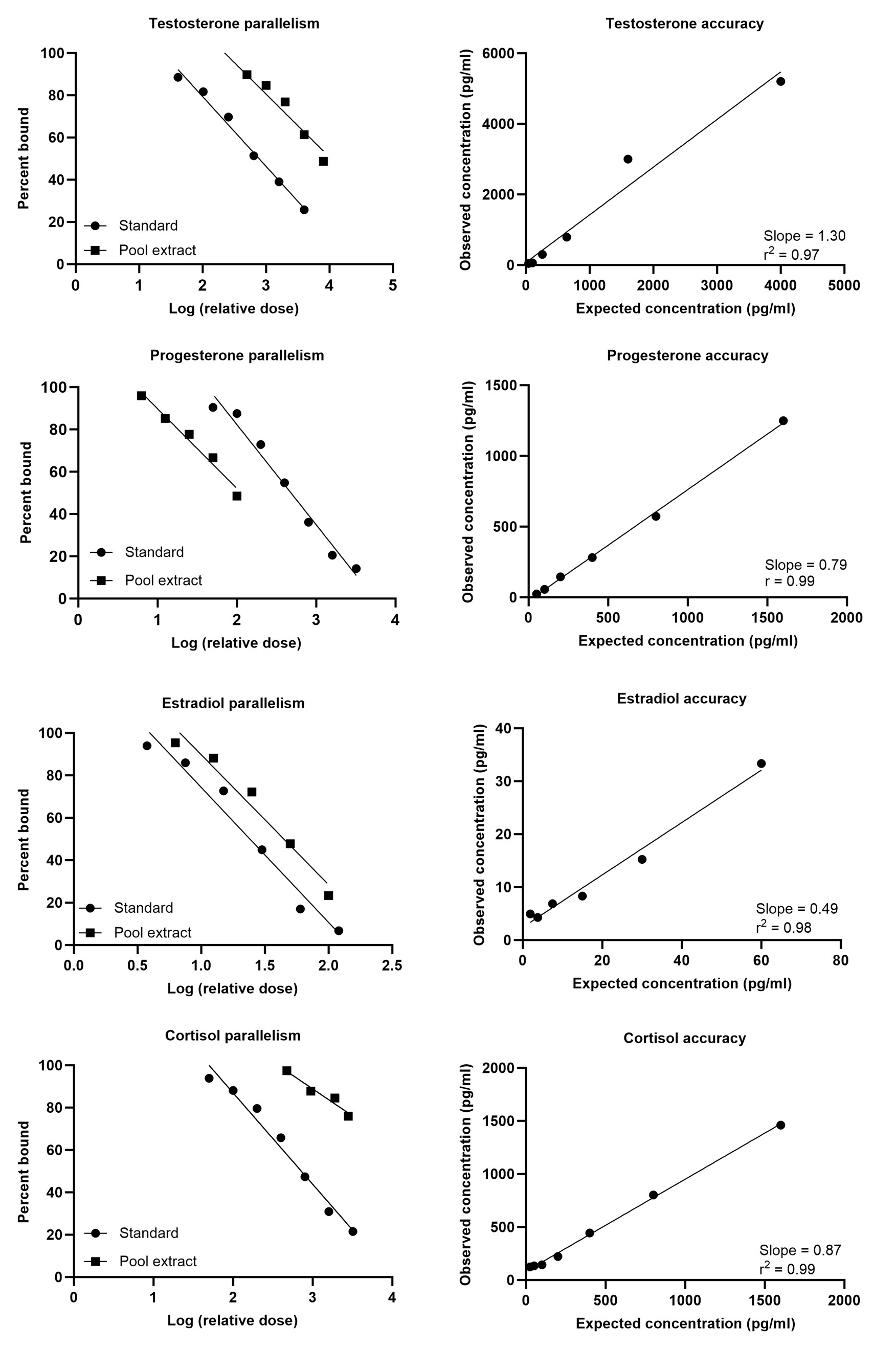

Supplement: S1 Fig — Dilutions with no detectable hormone are not shown. (JPG) [file pone.0319212.s001.jpg]
