# Supplementary material for: Comprehensive assessment of the physical and health features of the threatened Araguaian River dolphin Inia araguaiaensis
Source: PLoS One. 2025 Mar 31;20(3):e0319212. doi: 10.1371/journal.pone.0319212 (PMC11957337; doi:10.1371/journal.pone.0319212)
Supplement: S1 Table — (DOCX) [file pone.0319212.s002.docx]

| Variable | RBC (10^12^/L) | Hemoglobin (g/dL) | MCV (fL) | MCH (fmol) | MCHC (g/L) |
| --- | --- | --- | --- | --- | --- |
| Hct (L:L) | **0.62^b^** | **0.46^a^** | -0.04 | -0.34 | **-0.47^a^** |
| RBC (10^12^/L) |  | 0.27 | **-0.73^b^** | **-0.80^b^** | -0.27 |
| Hemoglobin (g/dL) |  |  | -0.02 | 0.26 | **0.42^b^** |
| MCV (fL) |  |  |  | **0.78^a^** | 0.09 |
| MCH (fmol) |  |  |  |  | **0.61^a^** |

Hct, hematocrit; RBC, red blood cell; MCV, mean corpuscular volume; MCH, mean corpuscular hemoglobin; MCHC, mean corpuscular hemoglobin concentration.

^a^ = Pearson, ^b^ = Spearmann
