# Supplementary material for: Comprehensive assessment of the physical and health features of the threatened Araguaian River dolphin Inia araguaiaensis
Source: PLoS One. 2025 Mar 31;20(3):e0319212. doi: 10.1371/journal.pone.0319212 (PMC11957337; doi:10.1371/journal.pone.0319212)
Supplement: S2 Table — (DOCX) [file pone.0319212.s003.docx]

| Variable | Monocyte (%) | Monocyte (10^9^/L) | Lymphocyte (%) | Lymphocyte (10^9^/L) | Basophil (%) | Basophil (10^9^/L) | Eosinophil (%) | Eosinophil (10^9^/L) | Neutrophil (%) | Neutrophil (10^9^/L) | Band neut (%) | Band neut (10^9^/L) |
| --- | --- | --- | --- | --- | --- | --- | --- | --- | --- | --- | --- | --- |
| WBC | **0.41^b^** | **0.72^b^** | 0.24 | **0.85^a^** | -0.2 | -0.20 | -0.01 | **0.61^a^** | -0.30 | **0.81^a^** | **-0.43^b^** | -0.20 |
| Monocyte (%) |  | **0.92^b^** | -0.0 | 0.31 | -0.03 | -0.03 | -0.01 | 0.17 | -0.13 | 0.28 | -0.08 | -0.01 |
| Monocyte (10^9^/L) |  |  | 0.15 | **0.64^a^** | -0.12 | -0.11 | -0.07 | **0.42^a^** | -0.32 | **0.49^a^** | -0.21 | -0.05 |
| Lymphocyte (%) |  |  |  | **0.70^a^** | -0.13 | -0.13 | -0.13 | 0.10 | **-0.94** | -0.31 | -0.06 | -0.09 |
| Lymphocyte (10^9^/L) |  |  |  |  | -0.28 | -0.27 | -0.04 | **0.52^a^** | **-0.73** | 0.40 | -0.40 | -0.19 |
| Basophil (%) |  |  |  |  |  |  | -0.27 | -0.36 | 0.23 | -0.04 | **0.42^b^** | **0.43^b^** |
| Basophil (10^9^/L) |  |  |  |  |  |  | -0.30 | -0.36 | 0.30 | 0.01 | **0.41^b^** | **0.44^b^** |
| Eosinophil (%) |  |  |  |  |  |  |  | **0.75^a^** | -0.11 | -0.08 | -0.17 | -0.18 |
| Eosinophil (10^9^/L) |  |  |  |  |  |  |  |  | -0.32 | 0.40 | -0.36 | -0.26 |
| Neutrophil (%) |  |  |  |  |  |  |  |  |  | 0.30 | -0.01 | 0.02 |
| Neutrophil (10^9^/L) |  |  |  |  |  |  |  |  |  |  | -0.24 | -0.001 |
| Band neut (%) |  |  |  |  |  |  |  |  |  |  |  | **0.94^b^** |

WBC, white blood cell count; Band neut, band neutrophils.

^a^ = Pearson, ^b^ = Spearmann
